# Effects of Heavy Strength Training on Running Performance and Determinants of Running Performance in Female Endurance Athletes

**DOI:** 10.1371/journal.pone.0150799

**Published:** 2016-03-08

**Authors:** Olav Vikmoen, Truls Raastad, Olivier Seynnes, Kristoffer Bergstrøm, Stian Ellefsen, Bent R. Rønnestad

**Affiliations:** 1 Section for Sport Science, Lillehammer University College, Lillehammer, Norway; 2 Department of Physical Performance, Norwegian School of Sport Sciences, Oslo, Norway; University of Rome Foro Italico, ITALY

## Abstract

**Purpose:**

The purpose of the current study was to investigate the effects of adding strength training to normal endurance training on running performance and running economy in well-trained female athletes. We hypothesized that the added strength training would improve performance and running economy through altered stiffness of the muscle-tendon complex of leg extensors.

**Methods:**

Nineteen female endurance athletes [maximal oxygen consumption (VO_2max_): 53±3 ml∙kg^-1^∙min^-1^, 5.8 h weekly endurance training] were randomly assigned to either normal endurance training (*E*, n = 8) or normal endurance training combined with strength training (*E+S*, n = 11). The strength training consisted of four leg exercises [3 x 4–10 repetition maximum (RM)], twice a week for 11 weeks. Muscle strength, 40 min all-out running distance, running performance determinants and patellar tendon stiffness were measured before and after the intervention.

**Results:**

*E+S* increased 1RM in leg exercises (40 ± 15%) and maximal jumping height in counter movement jump (6 ± 6%) and squat jump (9 ± 7%, p < 0.05). This was accompanied by increased muscle fiber cross sectional area of both fiber type I (13 ± 7%) and fiber type II (31 ± 20%) in *m*. *vastus lateralis* (p < 0.05), with no change in capillary density in *m*. *vastus lateralis* or the stiffness of the patellar tendon. Neither *E+S* nor *E* changed running economy, fractional utilization of VO_2max_ or VO_2max_. There were also no change in running distance during a 40 min all-out running test in neither of the groups.

**Conclusion:**

Adding heavy strength training to endurance training did not affect 40 min all-out running performance or running economy compared to endurance training only.

## Introduction

The effects of strength training on running performance has been examined in a number of studies with the majority reporting improved running performance [1–6]. However, the literature is far from conclusive, as some studies report no beneficial effect of strength training on running performance [7–10]. Running performance is mainly determined by the maximal oxygen consumption (VO_2max_), fractional utilization of VO_2max_ and running economy [11]. Addition of strength training has neither a negative nor a positive effect on VO_2max_ (e.g., [2, 6, 12]). The effect of combining strength and endurance training on fractional utilization of VO_2max_ has not been directly investigated, but the indirect measure of VO_2_ at the lactate threshold, expressed as percent of VO_2max_, seems to be unchanged [2, 12]. Running economy on the other hand seems to be positively affected by strength training (e.g., [2, 6, 12–14]). An improved running performance following strength training is therefore suggested to be mainly related to improved running economy [2, 6].

One of the most frequent proposed mechanisms behind improved running economy after strength training is changes in the stiffness of lower limb muscles and tendons [2, 14, 15]. During the first part of the contact phase in the running stride, elastic energy is stored in the muscles, tendons and ligaments acting across joints [16]. A partial return of this stored energy during the second part of the contact phase limits the muscle energy expenditure and amplifies the mechanical output of the muscle-tendon complex [16]. Hence, the stiffness of series elastic component, mainly tendons, can affect both the utilization of this elastic energy and the muscle contraction mechanics during the running stride. In fact, stiffer Achilles tendons have been associated with better running economy [17]. Intriguingly, more compliant patellar tendons were associated with better running economy [17], whereas heavy strength training has been shown to increase patellar tendon stiffness [18, 19]. A more compliant patellar tendon may indeed allow the muscle to operate at mechanically efficient lengths and velocities during the contact phase [17]. However, for a given tendon stiffness a stronger muscle would enable larger energy storage. Consequently, heavy strength training might induce changes in muscle and tendon properties with both potential beneficial and negative effects on running economy. It is therefore important to gain insight into the effects of strength training on patellar tendon mechanical properties, and if possible effects induces changes in running economy. However, to our best knowledge, no studies to date have investigated this.

Most research on the effects of strength training on running performance are performed with male athletes (e.g., [1, 3, 6, 15]) or a combination of male and female athletes (e.g., [2, 5, 7, 20]). Unfortunately, there is performed a substantial lower volume of research in this area using only female athletes [10, 13]. Therefore, there is a need for more research with female athletes. This is especially true regarding the effect of strength training induced changes in patellar tendon stiffness on running economy since it seems that male and female tendons may react differently to increased loading [21].

Even though strength training may enhance middle to long distance running performance through improved running economy it will also normally increase cross sectional area (CSA) of the muscle fibers [22]. Therefore, it can be speculated that strength training can increase diffusion distances from the capillaries to the interior of muscle cells, which will be negative for performance. In untrained individuals there are reports of increased or unchanged numbers of capillaries around each muscle fiber [23, 24] and no change in capillaries per fiber area [24] after strength training. However, as performing endurance training concurrently with strength training may blunt the hypertrophic response (e.g., [25]), and endurance trained athletes have larger numbers of capillaries than untrained [26, 27] these findings may not apply for endurance athletes. Consequently, there is a need to look closer into the effects of combined strength and endurance training on capillarization and fiber CSA in well-trained endurance athletes.

The purpose of this study was to investigate the effects of 11 weeks of heavy strength training on running performance during a 40 min all-out test and running economy in well-trained female endurance athletes. Furthermore, we wanted to assess the effects of the strength training on the mechanical properties of the patellar tendon to elucidate whether this could be related to changes in running performance and running economy. To investigate if strength training would have any effect on capillarization in endurance athletes we measured muscle fiber CSA and capillarization in *m*. *vastus lateralis*.

We hypothesized that the addition of heavy strength training would result in improved 40 min all-out performance and improved running economy and that these changes would be related to changes in mechanical properties of the patellar tendon, together with no negative effect on capillarization.

## Materials and Methods

### Ethical approval

The study was approved by the Local Ethics Committee at Lillehammer University College. Written informed consent was obtained from all athletes prior to inclusion, and the study was carried out in accordance with the Declaration of Helsinki.

### Participants

Twenty-eight female endurance athletes active in both cycling and running and that fulfilled at least two of Jeunkedrup et al.’s [28] training and race status descriptions of a well-trained endurance athlete were recruited to this study. None of the athletes had performed systematic strength training for the last 12 months leading up to the study (less than one session per week). The athletes were matched on VO_2max_ and randomly assigned to either adding heavy strength training to the ongoing endurance training (*E+S*, n = 14) or endurance training only (*E*, n = 14). During the study, three athletes in *E+S* left the project for reasons unrelated to the project protocol: one because of an injury, one because of a prolonged period of illness during the last part of the intervention and one because of other medical reasons. In *E*, six athletes left the study for reasons unrelated to the project protocol (injuries in training, pregnancy and lack of time). Therefore, the final numbers of athletes in *E+S* and *E* were 11 and 8, respectively.

### Experimental overview

This study is part of a larger study investigating the effects of heavy strength training on various aspects of endurance performance. Some of the basic characteristics as anthropometrics and endurance training have been reported previously [29].

The strength training program for the *E+S* group consisted of two strength training sessions per week and lasted for 11 weeks (during the competition period from April to July). Testing before and after the intervention period was performed over four test-days. During pre-tests, day one was utilized to sample muscle biopsies from the right *m*. *vastus lateralis*, and measure the mechanical properties of the left patellar tendon. At day two 1RM in one-legged leg press and half squat was measured. Day 3 consisted of an incremental running test for determination of blood lactate profile, a VO_2max_ test and testing of maximal squat jump (SJ) and counter movement jump (CMJ) height. Day 4 consisted of a 40 min all-out running test. There were at least 7 days between day one and two and 3–7 days between the remaining test-days. All tests for each participant were completed within 2–3 weeks. During post tests, athletes in *E+S* maintained their strength training with one session per week until all testing were complete. In general, post-tests were performed in the same order as pre-tests. However, muscle biopsies and patellar tendon measurements were moved to the last test day. The athletes did not perform any systematic periodization so neither the pre tests nor post tests were performed in a particular phase of periodization.

### Training

Endurance training duration and intensity were calculated based on heart rate (HR) recordings. Endurance training was divided into three HR zones: 1) 60%-82%, 2) 83%-87%, and 3) 88%-100% of maximal HR. The endurance training performed has been previously reported [29]. In brief, there were no significant differences between groups in the mean weekly duration of the endurance training, the distribution of this training within the three intensity zones (across groups values were: zone 1: 3.7 ± 1.6 h, zone 2: 1.1 ± 0.5 h, zone 3: 0.8 ± 0.5) and the numbers of endurance training sessions per week (across groups values were 4.3 ± 1 session · week^-1^).

The heavy strength training sessions for the *E+S* groups targeted leg muscles and were performed twice per week during the 11-week intervention period. Adherence to the strength training was high, with *E+S* athletes completing 21.4 ± 1.0 (range 19–22) of the planned 22 strength-training sessions. The strength-training program was performed as reported in Vikmoen et al. [29]. Briefly, each strength training session consisted of four leg exercises: half squat in a smith machine (Gym 80 International, Gelsenkirchen, Germany), leg press with one leg at a time (Gym 80 International, Gelsenkirchen, Germany), standing one-legged hip flexion in a cable cross machine (Gym 80 International, Gelsenkirchen, Germany), and ankle plantar flexion in a smith machine. For a detailed description of the exercises, see Ronnestad et al. [30]. Three sets were performed *per* exercise. An investigator supervised the athletes at all workouts during the first two weeks and at least one workout per week thereafter. During weeks one to three, athletes trained with 10RM sets at the first session and 6RM sets at the second session. These alternating loads were adjusted to 8RM and 5RM during weeks four to six, and was further adjusted to 6RM and 4RM during weeks seven to eleven (Table 1). The numbers of repetitions was always the same as the prescribed RM load meaning that the sets were performed until failure, and the athletes adjusted the absolute load as they got stronger to correspond to the prescribed RM load. The athletes were allowed assistance on the last repetition if necessary. Because a proposed mechanisms behind improved running performance after strength training is an increased rate of force development [2] the athletes were instructed to perform the concentric phase of the exercises with focus on maximal effort (duration around 1 s) while the eccentric phase was performed more slowly (duration 2–3 s). During each strength training session the athletes consumed a bar containing 15 g protein (Squeezy recovery bar, Squeezy Sports Nutrition, Braunschweig, Germany) to ensure adequate protein intake in conjunction with the strength training sessions. The athletes were encouraged to perform the strength training and endurance training on separate days. On days the athletes had to perform both endurance and strength training, they were encouraged to perform strength training in the first session of the day.

**Table 1:** Training loads used during the strength training intervention

Week	Load session 1	Load session 2
1–3	10RM	6RM
4–6	8RM	5RM
7–11	6RM	4RM

RM: Repetition maximum

### Strength, jumping and running tests

The athletes were instructed to refrain from intense exercise the day preceding testing, to prepare for the tests as they would have done for a competition, and to consume the same type of meal before each test. Running tests was performed on a motor driven treadmill (Woodway Desmo Evo, Waukesha, Wisconsin, USA). The inclination of the treadmill was set to 5.3% at all tests. All testing were performed under similar environmental conditions (18–20˚C).

#### 1RM tests

1RM was tested in one-legged leg press and half squat and the mean value from these two exercises were used for statistical analyses. Prior to the testing day, each athlete was given a supervised familiarization session to learn proper lifting technique, find individual equipment settings and practice SJ and CMJ. During this session, the load was gradually increased to allow estimation of a proper starting point for the 1RM testing.

The 1RM tests in both exercises were performed as previously described (Vikmoen et al. 2015). Briefly, testing started with a specific warm-up, consisting of three sets with gradually increasing load (40, 75 and 85% of expected 1RM) and decreasing number of repetitions (10→6→3). The first attempt was performed with a load approximately 5% below the expected 1RM. If a lift was successful, the load was increased by approximately 5%. The test was terminated when the athletes failed to lift the load in 2–3 attempts and the highest successful load lifted was noted as 1RM. Athletes were given 3 min rest between lifts.

#### Blood lactate profile

The blood lactate profile tests started with 5 min running at 7 km·h^-1^, which was subsequently increased every 5 min by 1 km·h^-1^. Between consecutive 5 min bouts there was a 1 min break, wherein blood was sampled from a finger-tip and analyzed for whole blood lactate concentration ([la^-^]) using a Lactate Pro LT-1710 analyzer (Arcray Inc., Kyoto, Japan), and the rating of perceived exertion (RPE) was recorded. The test was terminated when a [la^-^] of 4 mmol∙L^-1^ or higher was measured. VO_2_ and HR were measured during the last 3 min of each bout, and mean values were used for statistical analysis. VO_2_ was measured (30 s sampling time) using a computerized metabolic system with mixing chamber (Oxycon Pro, Erich Jaeger, Hoechberg, Germany). The gas analyzers were calibrated with certified calibration gases of known concentrations before every test. The flow turbine (Triple V, Erich Jaeger, Hoechberg, Germany) was calibrated before every test with a 3 l, 5530 series, calibration syringe (Hans Rudolph, Kansas City, USA). HR was recorded using a Polar S610i heart rate monitor (Polar, Kempele, Finland). From this incremental running test, the running velocity at 3.5 mmol∙L^-1^ [la^-^] was calculated for each athlete from the relationship between [la^-^] and running velocity using linear regression between data points. Running economy was determined by the mean VO_2_ at a running velocity of 10 km·h^-1^.

#### VO_2max_

After termination of the blood lactate profile test the athletes ran for 10 min at a freely chosen submaximal workload. The VO_2max_ test was then initiated with 1 min running at 8 km·h^-1^ and the speed was increased by 1 km·h^-1^ every minute until exhaustion. The athletes received strong verbal encouragement to run for as long as possible. VO_2_ was measured continuously, and VO_2max_ was calculated as the mean of the two highest 30 s VO_2_ measurements. The VO_2max_ test was considered valid when two or more of the following criteria were met: a plateau in VO2 was despite increased workload, a respiratory exchange ration above 1.1 and HR_peak_ ± 10 bpm pf the predicted maximal HR (220-age) [31]. Peak running performance during the test (V_max_) was calculated as the mean running velocity during the last 2 min of the incremental test. The highest HR recorded during the test was taken as HR_peak_ and immediately after the test blood [la^-^] and RPE were recorded.

#### SJ and CMJ

Twenty min after termination of the VO_2max_ test, explosive strength was tested as maximal jumping height in SJ and CMJ. These jumps were performed on a force plate (SG-9, Advanced Mechanical Technologies, Newton, MA, USA, sampling frequency of 1kHz). After 3–5 submaximal warm up jumps, the athletes performed three SJ and three CMJ with 2–3 min rest between jumps. The highest SJ and CMJ were utilized for statistical analyses. During all jumps the athletes were instructed to keep their hands placed on their hips and aim for maximal jumping height. The SJ was performed from ~90 degrees knee angle. In this position, they paused for 3 s before jumping. No downward movement was allowed prior to the jump and the force curves were inspected to verify this. During the eccentric phase of the CMJ the athletes were instructed to turn at a knee angle they felt was optimal for achieving maximal jumping height.

#### 40 min all-out test

Prior to the 40 min all-out test, athletes performed 10 min warm up at self-selected submaximal velocities, containing three submaximal sprints performed during the last 2 min. These sprints were standardized from pre to post in each athlete. During the first 5 min of the test, the investigators set the velocity. This individual selected velocity was based on the lactate profile test and corresponded to the velocity at 2.5 mmol∙L^-1^ [la^-^]. Thereafter, running velocity were controlled by the athletes themselves, with instructions to run the greatest distance possible during the 40 min. Measurements of VO_2_ was made during the last minute of every 5 min section, to allow estimation of performance VO_2_ and fractional utilization of VO_2max_,. During this minute, athletes were not allowed to adjust the running velocity. The mean VO_2_ during this minute was estimated to reflect the mean VO_2_ during the corresponding 5 min section. During the last 5 min of the test, VO_2_ was measured continuously as pilot testing showed that athletes performed numerous velocity adjustments during this part of the test. Performance VO_2_ was calculated as the mean VO_2_ of all 5 min sections, and fractional utilization of VO_2max_ was calculated as performance VO_2_ in percentage of VO_2max_. During the test, the athletes were allowed to drink water *ad libitum*.

### Measurements of the mechanical and material properties of the patellar tendon

All the measurements of the mechanical and material properties of the patellar tendon were performed on the left leg and were done as previously described [32]. Briefly, the athletes were seated with a 90° angle in both knee and hip joint in a knee extension apparatus (Knee extension, Gym 2000, Geithus, Norway) instrumented with a force cell (U2A, Hottinger Baldwin Messtechnik GmbH, Darmstadt, Germany). To measure patellar tendon CSA, transversal scans were performed proximally, medially and distally along the tendon length using an B-mode ultrasound apparatus (HD11XE, Phillips, Bothell, WA, USA). Sagittal scanning was used to measure tendon length. To measure tendon force and elongation the ultrasound probe was attached to the left knee with a custom-made device. The athletes performed ramp contractions at a constant rate of 100 N·s^-1^. To correct for hamstring co-activation when calculating tendon force (see below), a maximal isometric knee flexion were performed after the knee extension test. In addition, EMG data were recorded (TeleMyo 2400 G2 telemetry Systems, Noraxon Inc., Scottsdale, AZ, USA) from the biceps femoris muscle during isometric knee extension and flexion. Patellar tendon force (F_PT_) was calculated as the force measured in the force cell, corrected for hamstring co-activation, internal and external moment arms as follows:

$$F_{PT}=((F_{q}+F_{h})M_{e})/M_{i}$$

Where F_q_ is force measured by the force cell, F_h_ is estimated hamstrings co-activation force, M_i_ and M_e_ corresponds to internal and external moment arm respectively.

*Tendon morphology data* were analysed as previously described [32], using an image analysis software (ImageJ 1.45s, National Institute of Health, Austin, TE, USA). Tendon elongation data were analyzed using a video analysis software (Tracker Video Analysis and Modeling Tool, Open Source Physics, Douglas Brown, 2012). The patellar apex and the tibia plateau were digitally marked within a common coordinate system. The actual elongation of the tendon was calculated as the change in the distance between coordinates of these anatomical landmarks. To calculate tendon material and mechanical properties force-elongation curves were fitted with a 2nd degree polynomial. All the recordings used in the results had a fit of R^2^ = 0,92 or higher. Stiffness was calculated as the slope of the force–elongation curve, between 90 and 100% of each athlete’s maximal force. The Young’s modulus was calculated by multiplying the stiffness values by the ratio between the patellar tendon resting length (l_0_) and mean CSA. Patellar tendon l_0_ and maximal length (l_max_) was used to calculate the patellar tendon strain. Two sets of ultrasound data (from two *E+S* athletes) had to be discarded because of an insufficient quality to enable analysis. Therefore, the number of athletes included in the data from tendon testing is 9 in *E+S* and 8 in *E*.

### Muscle biopsy sampling

Muscle biopsies were sampled from *m*. *vastus lateralis* using the Bergström procedure. Athletes were instructed to refrain from physical activity for the last 24h leading up to biopsy sampling. During each biopsy sampling-event, two separate muscle biopsies were retrieved and pooled in a petri dish filled with sterile physiological salt water. An appropriately sized muscle sample (mean wet weight: 29 ± 8 mg) was selected for immunohistochemical analyses and mounted in Tissue-Tek (Sakura Finetek USA, Inc., Torrance, CA, USA) and quickly frozen in isopentane cooled on liquid nitrogen. Muscle samples were stored at– 80°C until later analyses.

### Immunohistochemistry

Cross-sections 8 *μ*m thick were cut using a microtome at −20°C (CM3050; Leica Microsystems GmbH, Wetzlar, Germany) and mounted on microscope slides (Superfrost Plus; Thermo Fisher Scientific, Inc., Waltham, MA, USA). The sections were then air-dried and stored at −80°C. Prior to antibody labelling, muscle sections were blocked in a solution containing 1% BSA (cat. no. A4503; Sigma-Aldrich Corp., St Louis, MO, USA) and 0.05% PBS-T solution (cat. no. 524650; Calbiochem, EMD Biosciences, Inc., San Diego, CA, USA) for 30 min. Then they were incubated overnight at 4°C with antibodies against the capillary marker CD31 (1:200; clone JC70A, M0823; Dako A/S, Glostrup, Denmark), followed by incubation with appropriate secondary antibodies (Alexa Fluor, cat. no. A11005).

Following staining, muscle sections were visualized and pictures taken using a high-resolution camera (DP72; Olympus Corp., Tokyo, Japan) mounted on a microscope (BX61; Olympus Corp.) with a fluorescence light source (X-Cite 120PCQ; EXFO Photonic Solutions Inc., Mississauga, Ontario, Canada).

These muscle sections were then incubated for 1 hour at room temperature with antibodies against myosin heavy chain type II (1:1000; SC71; gift from Professor S. Schiaffino) and dystrophin (1:1000; cat. no. ab15277; Abcam Plc), followed by incubation with appropriate secondary antibodies (Alexa Fluor, cat. no. A11005 or A11001; Invitrogen, Inc.).

Muscle sections were then covered with a coverslip and glued with ProLong Gold Antifade Reagent with DAPI (cat.no. P36935; Invitrogen Molecular Probes, Eugene, OR,USA) and left to dry overnight at room temperature. Muscle sections were again visualized and new pictures was taken at the exactly same location in the section as the CD31 picture. Between all stages, sections were washed for 3 × 5 min using a 0.05% PBS-T solution.

Fiber type distribution, fiber cross-sectional area and capillaries were identified using TEMA software (CheckVision, Hadsund, Denmark). Capillarization was expressed as capillaries around each fiber (CAF) and capillaries related to fiber area (CAFA), for type I and type II (IIA and IIX) fibers. Because of technical problems with some analyses, the number of athletes in the immunohistochemistry data is 8 in *E+S* and 5 in *E*.

### Statistical analyses

All data in the text, Figs and tables are presented as mean ± standard deviation, unless otherwise stated. Data were analyzed using two-way (group x time) repeated measures ANOVA. Effect sizes (ES) were calculated for key performance and physiological adaptations to elucidate on the practical significance of strength training. ES were calculated as Cohen’s d and the criteria to interpret the magnitude were the following: 0–0.2 = trivial, 0.2–0.6 = small, 0.6–1.2 = moderate, 1.2–2.0 = large and ˃ 2 = very large [33].

Correlations analyses were done using the Pearson product-moment method. Analyses was performed in Excel 2013 (Microsoft Corporation, Redmon, WA, USA). Analyses resulting in p ≤ 0.05 were considered statistically significant.

## Results

There were no significant differences between *E+S* and *E* in any of the measured variables at baseline.

### Body mass, maximal leg strength and muscle fiber area

Body mass remained unchanged in *E+S* (from 62.4 ± 5.2 kg to 63.1 ± 5.6 kg) but was slightly reduced in *E* (from 65.6 ± 8.4 kg to 64.8 ± 8.0 kg, p < 0.05). There was a significant interaction (p < 0.05) between group and time (pre vs post) indicating that the change in body mass was different between the groups.

**Fig 1:**
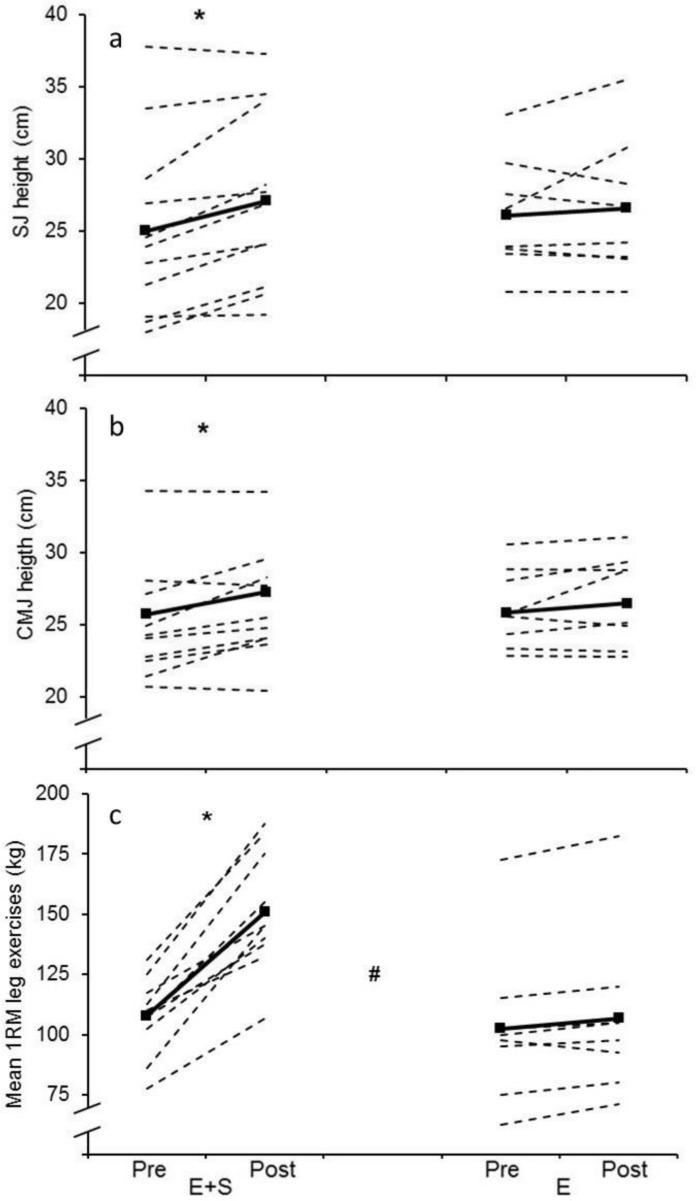
Maximal strength and jumping performance. Individual values (dotted lines) and mean values (solid lines) before (Pre) and after (Post) the intervention period for athletes adding strength training to their normal endurance training (*E+S*) and athletes performing normal endurance training only (*E*). a: Squat jump (SJ) height. b: Counter movement jump (CMJ) height. c: Mean 1 repetition maximum (1RM) in half-squat and one-legged leg press (leg exercises). * Different from pre (p ˂ 0.05), # significant interaction between group and time (p ˂ 0.05)

1RM in the leg exercises increased 40.4 ± 14.7% in *E+S* (p < 0.01, Fig 1) with no change in *E*. There was a significant interaction (p < 0.01) between group and time (pre vs post). In addition, the effect size analyses revealed a very large practical effect of *E+S* compared to *E* (ES = 3.20).

**Fig 2:**
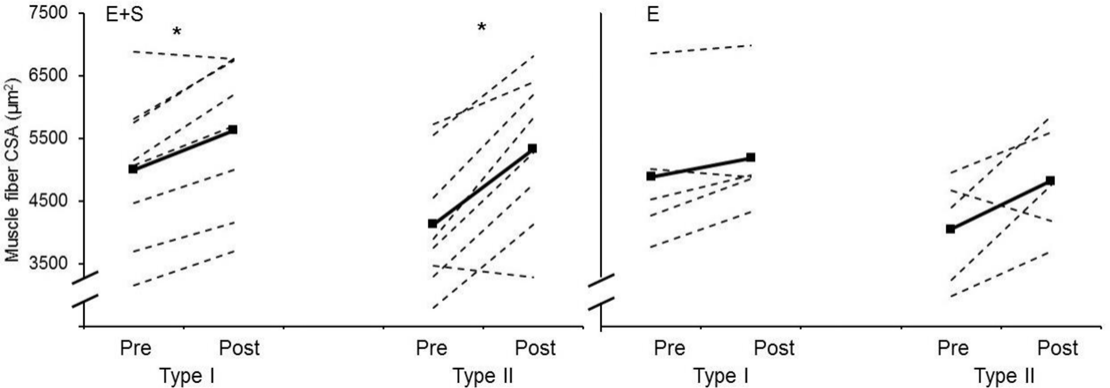
Muscle fiber cross sectional area. Individual values (dotted lines) and mean values (solid lines) before (Pre) and after (Post) the intervention period for athletes adding strength training to their normal endurance training (*E+S*, left panel) and athletes performing normal endurance training only (*E*, right panel). Muscle fiber cross sectional area (CSA) for both type I muscle fibers and type II muscle fibers * Different from pre (p ˂ 0.05)

In *E+S*, CSA of both type I and type II muscle fibers increased in *m*. *vastus lateralis* (13.2 ± 6.8% and 30.8 ± 19.6%, respectively, p < 0.01), with no changes occurring in *E* (Fig 2). *E+S* had a moderate practical effect on muscle fiber CSA compared to *E* (ES = 0.83).

### SJ and CMJ

*E+S* increased SJ and CMJ height by 8.9 ± 6.8% and 5.9 ± 6.0% respectively (p ˂ 0.05) while no changes occurred in *E* (Fig 1). The effect size analyses revealed a moderate practical effect in favor of *E+S* in both SJ (ES = 1.06) and CMJ (ES = 0.65).

### Capillarization

**Fig 3:**
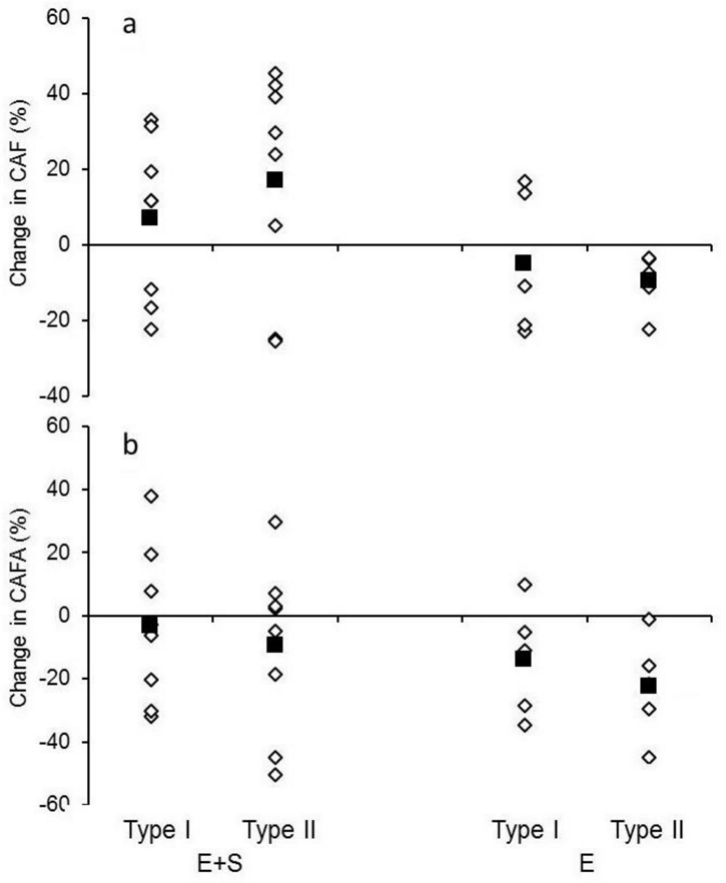
Capillarization. Individual values (open symbols) and mean values (solid squares) for athletes adding strength training to their normal endurance training (*E+S*) and athletes performing normal endurance training only (*E*). a: Percent change in capillaries around each muscle fiber (CAF) for both muscle fiber type I and muscle fiber type II for *E+S* and *E*. b: Percent change in capillaries related to fiber area (CAFA) for both muscle fiber type I and muscle fiber type II for *E+S* and *E*.

None of the groups had any change in CAF or CAFA around neither type I nor type II fibers (Fig 3).

### Mechanical and material properties of the patellar tendon

There were no significant changes in stiffness or Young’s modulus of the patellar tendon in neither *E+S* nor *E* (Table 2). The mean CSA of the patellar tendon increased by 5.2 ± 3.6% in *E+S* (p ˂ 0.01) while no significant changes occurred in *E* (Table 2).

**Table 2:** Stiffness, Young’s modulus and mean cross section area (CSA) of the patellar tendon.

	*E+S*		*E*	
	Pre	Post	Pre	Post
Stiffness (N·mm^-1^)	2752 ± 402	2483 ± 733	2753 ± 947	2692 ± 697
Young’s Modulus (MPa)	1038 ± 194	925 ± 162	1251 ± 296	1158 ± 273
Mean CSA (mm^2^)	65.9 ± 7.1	69.2 ± 6.9*	60.3 ± 4.2	59.9 ± 4.4

Stiffness, Young’s modulus and mean cross section area (CSA) of the patellar tendon before (Pre) and after (Post) the intervention period for athletes adding strength training to their normal endurance training (*E+S*) and athletes performing normal endurance training only (*E*). Values are mean ± SD

* Different from pre (p ˂ 0.05).

### VO_2max_ and V_max_

Both VO_2max_ and V_max_ was unchanged in both groups during the intervention period (Table 3).

**Table 3:** Data from the maximal oxygen consumption test.

	*E+S*		*E*	
	Pre	Post	Pre	Post
VO_2max_ (ml∙kg^-1^∙min^-1^)	52.2 ± 2.3	52.7 ± 3.3	54.2 ± 2.9	53.1 ± 1.9
V_max_ (km·h^-1^)	12.8 ± 0.7	13.0 ± 0.9	13.1 ± 0.5	13.3 ± 0.6
HR_peak_ (beats∙min^-1^)	193 ± 9	192 ± 9	189 ± 8	187 ± 7
RPE	19 ± 1	20 ± 1	19 ± 1	19 ± 1
[La^-1^]_peak_ (mmol∙l^-1^)	9.7 ± 3.0	8.1 ± 3.8	8.9 ± 2.2	7.7 ± 1.8

Data from the maximal oxygen consumption (VO_2max_) test before (Pre) and after (Post) the intervention period for athletes adding strength training to their normal endurance training (*E+S*) and athletes performing normal endurance training only (*E*). Values are mean ± SD.

### Running economy and running velocity at 3.5 mmol∙L^-1^ [la^-^]

**Fig 4:**
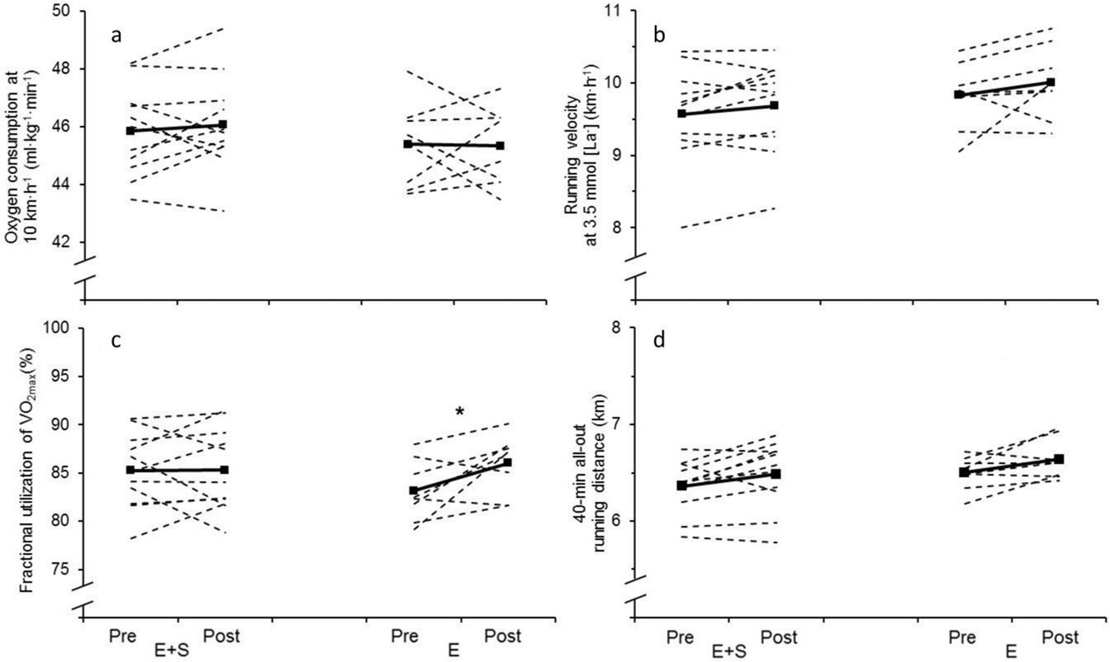
Determinants of running performance and running performance. Individual values (dotted lines) and mean values (solid lines) before (Pre) and after (Post) the intervention period for athletes adding strength training to their normal endurance training (*E+S*) and athletes performing normal endurance training only (*E*). a: Body mass adjusted oxygen consumption at 10 km·h^-1^. b: Running velocity at 3.5 mmol∙L^-1^ [la^-^] calculated during the blood lactate profile test. c: The fractional utilization of VO_2max_ during the 40 min all-out test. d: Running distance during the 40 min all-out test. * Different from pre (p ˂ 0.05).

There were no changes in running economy measured at 10 km**∙**h^-1^ during the blood lactate profile test (Fig 4) or running velocity at 3.5 mmol∙L^-1^ [la^-^] (Fig 4) in neither of the groups.

### 40 min all-out test

There were no change in running distance or performance VO_2_ during the 40 min all-out test in neither of the groups during the intervention (Fig 4). Fractional utilization of VO_2max_ did not change in *E+S* (from 85.3 ± 3.9 to 85.3 ± 4.3, Fig 4), but increased in *E*, going from 83.2 ± 3.1% to 86.0 ± 3.0% (p ˂ 0.05).

Before the intervention the performance in the 40 min all-out test correlated with velocity at 3.5 mmol∙L^-1^ [la^-^], VO_2max_ and V_max_ (r = 0.65, r = 0. 58, r = 0.79, respectively), but not with running economy (r = -0.24). No significant correlations between changes in these variables and changes in 40 min all-out running distance were observed.

## Discussion

The main results from the current study were that adding heavy strength training to well-trained female athletes`normal endurance training did not affect the mechanical properties of the patellar tendon or running economy. Furthermore, there was no effect on running performance during a 40 min all-out running test. Strength training had no negative effect on capillary density despite increased muscle fiber CSA and muscle strength.

### Maximal strength and muscle fiber cross sectional area

The strength-training program used in the current study was effective in increasing maximal leg strength as shown by an increase in 1RM in the leg exercises. This is in accordance with previously observed increases in 1RM in endurance athletes adding heavy strength training to their normal endurance training (e.g., [2, 4, 13]). The results from the current study confirms previous results [2, 34] that a quite large increase in muscular strength can be achived without an increased body mass. This is important for runners since increased body mass can negatively influence running performance. In spite of this, the improved strength seemed to be at least partially dependent on increased muscle mass, as evident from the increased muscle fiber CSA. The present muscle hypertrophy is in agreement with other studies using similar strength training protocols in endurance athletes [34–36]. Interestingly, there were no difference in the CSA of the type I and type II fibers in the current athletes, confirming the notion that in endurance athletes the type I fibers may be just as large [37] or even larger [38] than the type II fibers.

### SJ and CMJ

The current strength training protocol was also effective in increasing leg muscle power, as evident from the increased SJ and CMJ performance. This is in line with previous reports of effects of heavy strength training on jumping ability in untrained participants [39, 40]. However, previous data from endurance athletes are more unclear, as some studies report improved jumping performance [36, 41] whereas others do not [14, 20]. The current study indicate that quite large improvements in jumping ability and explosive strength can be achived with heavy strength traning despite concurrently performing endurance training.

### Capillarization

Eleven weeks of heavy strength training did not affect capillarization expressed as either CAF or CAFA, despite leading to significant muscle fiber hypertrophy. Importantly, this suggest that the potentially negative effect of increased muscle fiber CSA on diffusion distances between blood and inner parts of muscle fibers was counteracted by a non-significant increase in CAF. However, this data should be treated with caution because of the limited sample size. Despite of this, our finding are in line with previous studies in untrained participants that have reported either no change or a slight increase in CAF [23, 24] and no change in CAFA [24] after a period of heavy strength training. Our finding is also in agreement with results reported in elite male cyclists after 16 weeks of heavy strength training [35]. Therefore, it seems like endurance athletes should not be afraid of reduced capillarization when they consider adding heavy strength training to their ongoing endurance training.

### Mechanical properties of the patellar tendon

The lack of changes in mechanical properties of the patellar tendon following heavy strength training is in contrast to most studies, typically reporting increased patellar tendon stiffness, at least in previously untrained participants [19, 42–44]. A possible reason for the discrepancy in results between our study and that by others is that we included female participants while the other studies included males [19, 42–44]. In fact, female tendons have been reported to show a lower rate of new connective tissue formation in response to mechanical loading [21]. Differences in the strength training protocol may also explain the lack of changes in the current study. Indeed, most previous studies reporting increased patellar tendon stiffness following strength training have included heavy knee extension exercise [19, 42, 44] or isometric muscle actions [45]. In the current study, the exercises involved were more complex involving multiple joints that perhaps reduced the absolute mechanical loading on the patellar tendon compared to a pure knee extension exercise. In addition, the athletes were instructed to perform the concentric phase of the exercises as fast as possible making the time under tension quite low.

In contrast to the lack of effect of strength training on patellar tendon properties, it led to increases in its CSA. In line with these findings, some studies on the effect of strength training, yet not all [18], reports an increase in patellar tendon CSA [19, 42]. Without changes in mechanical properties, the tendon hypertrophy measured here suggests that material properties may also have been altered. The lack of change in Young’s modulus following training may highlight the limitation of this parameter based on finite tendon sections to reflect whole tendon material properties. Interpreting the mechanisms driving tendon hypertrophy extends beyond the scope of the present article. One could speculate that increasing tendon CSA may shield this tissue against damage caused by excessive and/or unusual stresses. Taken together, the present measurements indicate that resistance training triggers an adaptive response in the patellar tendon of female runners, without affecting the mechanical properties of this tissue. Whether this adaptation may affect injury rates or have other effects amongst runners warrants further investigation.

### VO_2max_, fractional utilization of VO_2max_, running velocity at 3.5 mmol·L^-1^ blood [La^-^], and running economy

The lack of change in VO_2max_ after strength training is not surprising and is actually in accordance with the current literature (e.g., [6, 12, 46]). Fractional utilization of VO_2max_ measured during the 40 min all-out test did not change in *E+S* during the course of the study. To our knowledge, this is the first study directly measuring fractional utilization of VO_2max_ in running after addition of heavy strength training in endurance athletes. However, VO_2_ at lactate threshold in percentage of VO_2max_, is often taken as an indirect measure of fractional utilization of VO_2max_ [11]. The few studies measuring this variable in running reports no effect after addition of heavy strength training [2, 12]. Notably, there was a slight increase in fractional utilization of VO_2max_ in *E* over the course of the intervention. This was likely due to a combination of two factors; a small but non-significant reduction in VO_2max_, largely due to one athlete exhibiting a large reduction, and a small but non-significant increase in performance VO_2_.

Surprisingly, we found no effect of heavy strength training on running economy, contrasting the majority of previous studies, typically reporting improvements from 3–8% [2, 6, 13, 14]. However, some studies supports the lack of an effect of strength training on running economy [1, 7, 8, 47]. In two of this studies [7, 47] the lack of improved running economy might be because the strength training program only consisted of one session for the legs per week.

Supporting the lack of an effect on VO_2max,_ fractional utilization of VO_2max_ and running economy, strength training had no effect on running velocity at 3.5 mmol·L^-1^ blood [La^-^]. The latter is in accordance with the majority of the current literature which reports no change in velocity at a certain blood [la^-1^] or ventilatory threshold after adding various forms of strength training to runners’ normal training [2, 7, 12], although exceptions exist [14]. This is quite surprising considering that improved running economy in theory should affect the running speed at a certain lactate threshold [11].

### Running performance

The lack of changes in 40 min all-out performance is not in line with many of the studies in this area where improved running performance have been reported [1, 2, 4–6]. However, no change in performance is in line with the present lack of changes in the important performance determining factors like VO_2max_, running economy and fractional utilization of VO_2max_. Since strength training does not affect VO_2max_ and the fractional utilization of VO_2max_, the mechanism for the observations of improved running performance in some other studies seems to be improved running economy [2, 6]. However, not all studies have found strength training to be beneficial for running performance [7, 8, 10, 47], and are in accordance with the current study. Interestingly, these studies do also report no improvements in running economy. Therefore, the lack of improved running performance in the current study is probably because of no changes in running economy.

Whilst unclear, the discrepancies in training-induced running performance measures between the current study and that by others may be attributed to methodological differences. In the current study, all running tests were performed at 5.3% inclination. This inclination resulted in a quite low running velocity compared to some other studies. Indeed, changes in running economy after strength training have previously been found to be related to running velocity [46]. However, improvements in running economy after strength training have also been reported at similar velocities [20, 36] and at the same inclination [48] used in the current study. Therefore, the inclination used is probably not the only explanation why no changes in running economy and performance were observed.

The fact that this study includes only female athletes while most previous studies include either only males or a mix of both male and female runners this may perhaps explain why no effects of strength training on running economy was observed. However, strength training have been reported to improve running economy in female runners [13] making this explanation unlikely.

One of the most frequent proposed mechanism for the possible ergogenic effect of strength training on running economy is changes in stiffness of the muscle or tendons in the legs [2, 14]. Despite this speculation, studies have yet to investigate the effect of heavy strength training on patellar tendon mechanical properties in conjunction with running economy. In the current study, the unchanged tendon stiffness and increased strength suggest that more elastic energy may be stored in the patellar tendon during the stance phase, amplifying muscular power output and efficiency. However, the lack of changes in running economy do not support this hypothesis, and conclusion cannot be drawn regarding the influence of patellar tendon properties in the present study.

V_max_ has been reported to be the best laboratory measure to predict performance in various running distances [49] and can actually be considered a measure of running performance [50]. The lack of increased V_max_ further indicates that heavy strength training did not lead to improved running performance in the current study. It has previously been reported both improved [6, 36] and no change [14] in V_max_ after heavy strength training in trained runners.

## Conclusion

In contrast to our hypothesis, adding heavy strength training to endurance training in well-trained female endurance athletes did not affect running performance measured as running distance during a 40 min all-out test. The lack of effect on performance was probably because the strength training intervention did not improve running economy or changed the mechanical properties of the patellar tendon. However, strength training had no negative effect capillary density.

## References

[1] Damasceno MV, Lima-Silva AE, Pasqua LA, Tricoli V, Duarte M, Bishop DJ, et al Effects of resistance training on neuromuscular characteristics and pacing during 10-km running time trial. Eur J Appl Physiol. 2015; 10.1007/s00421-015-3130-z 25697149

[2] Storen O, Helgerud J, Stoa EM, Hoff J. Maximal strength training improves running economy in distance runners. Med Sci Sports Exerc. 2008;40(6): 1087–1092. 10.1249/MSS.0b013e318168da2f 18460997

[3] Paavolainen L, Hakkinen K, Hamalainen I, Nummela A, Rusko H. Explosive-strength training improves 5-km running time by improving running economy and muscle power. J Appl Physiol. 1999;86(5): 1527–1533. 10233114 10.1152/jappl.1999.86.5.1527

[4] Hickson RC, Dvorak BA, Gorostiaga EM, Kurowski TT, Foster C. Potential for strength and endurance training to amplify endurance performance. J Appl Physiol. 1988;65(5): 2285–2290. 3209573 10.1152/jappl.1988.65.5.2285

[5] Barnes KR, Hopkins WG, McGuigan MR, Northuis ME, Kilding AE. Effects of resistance training on running economy and cross-country performance. Med Sci Sports Exerc. 2013;45(12): 2322–2331. 10.1249/MSS.0b013e31829af603 23698241

[6] Sedano S, Marin PJ, Cuadrado G, Redondo JC. Concurrent training in elite male runners: the influence of strength versus muscular endurance training on performance outcomes. J Strength Cond Res. 2013;27(9): 2433–2443. 10.1519/JSC.0b013e318280cc26 23287831

[7] Ferrauti A, Bergermann M, Fernandez-Fernandez J. Effects of a concurrent strength and endurance training on running performance and running economy in recreational marathon runners. J Strength Cond Res. 2010;24(10): 2770–2778. 10.1519/JSC.0b013e3181d64e9c 20885197

[8] Roschel H, Barroso R, Tricoli V, Batista MA, Acquesta FM, Serrao JC, et al Effects of strength training associated with whole body vibration training on running economy and vertical stiffness. J Strength Cond Res. 2015; 10.1519/JSC.0000000000000857 25627640

[9] Bertuzzi R, Pasqua LA, Bueno S, Damasceno MV, Lima-Silva AE, Bishop D, et al Strength-training with whole-body vibration in long-distance runners: a randomized trial. Int J Sports Med. 2013;34(10): 917–923. 10.1055/s-0033-1333748 23559412

[10] Kelly CM, Burnett AF, Newton MJ. The effect of strength training on three-kilometer performance in recreational women endurance runners. J Strength Cond Res. 2008;22(2): 396–403. 10.1519/JSC.0b013e318163534a 18550953

[11] Bassett DR, Howley ET. Limiting factors for maximum oxygen uptake and determinants of endurance performance. Med Sci Sports Exerc. 2000;32(1): 70–84. 10647532 10.1097/00005768-200001000-00012

[12] Millet GP, Jaouen B, Borrani F, Candau R. Effects of concurrent endurance and strength training on running economy and.VO(2) kinetics. Med Sci Sports Exerc. 2002;34(8): 1351–1359. 12165692 10.1097/00005768-200208000-00018

[13] Johnston RE, Quinn TJ, Kertzer R, Vroman NB. Strength training in female distance runners: Impact on running economy. J Strength Cond Res. 1997;11(4): 224–229.

[14] Guglielmo LG, Greco CC, Denadai BS. Effects of strength training on running economy. Int J Sports Med. 2009;30(1): 27–32. 10.1055/s-2008-1038792 18975259

[15] Spurrs RW, Murphy AJ, Watsford ML. The effect of plyometric training on distance running performance. Eur J Appl Physiol. 2003;89(1): 1–7. 12627298 10.1007/s00421-002-0741-y

[16] Roberts TJ, Azizi E. Flexible mechanisms: the diverse roles of biological springs in vertebrate movement. J Exp Biol. 2011;214(Pt 3): 353–361. 10.1242/jeb.038588 21228194 PMC3020146

[17] Arampatzis A, De Monte G, Karamanidis K, Morey-Klapsing G, Stafilidis S, Bruggemann GP. Influence of the muscle-tendon unit's mechanical and morphological properties on running economy. J Exp Biol. 2006;209(Pt 17): 3345–3357. 16916971 10.1242/jeb.02340

[18] Reeves ND, Maganaris CN, Narici MV. Effect of strength training on human patella tendon mechanical properties of older individuals. J Physiol. 2003;548(Pt 3): 971–981. 12626673 10.1113/jphysiol.2002.035576 PMC2342903

[19] Seynnes OR, Erskine RM, Maganaris CN, Longo S, Simoneau EM, Grosset JF, et al Training-induced changes in structural and mechanical properties of the patellar tendon are related to muscle hypertrophy but not to strength gains. Journal of applied physiology. 2009;107(2): 523–530. 10.1152/japplphysiol.00213.2009 19478195

[20] Francesca PM, Giulia DI, Stefania C, Alessandro S, Gianluca V, Antonio LT. Concurrent strength and endurance training effects on running economy in master endurance runners. J Strength Cond Res. 2012; 10.1519/JSC.0b013e3182794485 23207882

[21] Magnusson SP, Hansen M, Langberg H, Miller B, Haraldsson B, Westh EK, et al The adaptability of tendon to loading differs in men and women. International journal of experimental pathology. 2007;88(4): 237–240. 17696904 10.1111/j.1365-2613.2007.00551.x PMC2517312

[22] Folland JP, Williams AG. The adaptations to strength training: morphological and neurological contributions to increased strength. Sports Med. 2007;37(2): 145–168. 17241104 10.2165/00007256-200737020-00004

[23] Bell GJ, Syrotuik D, Martin TP, Burnham R, Quinney HA. Effect of concurrent strength and endurance training on skeletal muscle properties and hormone concentrations in humans. Eur J Appl Physiol. 2000;81(5): 418–427. 10751104 10.1007/s004210050063

[24] Hather BM, Tesch PA, Buchanan P, Dudley GA. Influence of eccentric actions on skeletal muscle adaptations to resistance training. Acta physiologica Scandinavica. 1991;143(2): 177–185. 1835816 10.1111/j.1748-1716.1991.tb09219.x

[25] Kraemer WJ, Patton JF, Gordon SE, Harman EA, Deschenes MR, Reynolds K, et al Compatibility of high-intensity strength and endurance training on hormonal and skeletal muscle adaptations. J Appl Physiol. 1995;78(3): 976–989. 7775344 10.1152/jappl.1995.78.3.976

[26] Ingjer F, Brodal P. Capillary supply of skeletal muscle fibers in untrained and endurance-trained women. Eur J Appl Physiol Occup Physiol. 1978;38(4): 291–299. 668683 10.1007/BF00423112

[27] Brodal P, Ingjer F, Hermansen L. Capillary supply of skeletal muscle fibers in untrained and endurance-trained men. The American journal of physiology. 1977;232(6): H705–712. 879309 10.1152/ajpheart.1977.232.6.H705

[28] Jeukendrup AE, Craig NP, Hawley JA. The bioenergetics of World Class Cycling. Journal of science and medicine in sport / Sports Medicine Australia. 2000;3(4): 414–433. 11235007 10.1016/s1440-2440(00)80008-0

[29] Vikmoen O, Ellefsen S, Troen O, Hollan I, Hanestadhaugen M, Raastad T, et al Strength training improves cycling performance, fractional utilization of VO and cycling economy in female cyclists. Scand J Med Sci Sports. 2015; 10.1111/sms.12468 25892654

[30] Ronnestad BR, Hansen EA, Raastad T. In-season strength maintenance training increases well-trained cyclists' performance. Eur J Appl Physiol. 2010;110(6): 1269–1282. 10.1007/s00421-010-1622-4 20799042

[31] Howley ET, Bassett DR Jr., Welch HG. Criteria for maximal oxygen uptake: review and commentary. Med Sci Sports Exerc. 1995;27(9): 1292–1301. 8531628

[32] Helland C, Bojsen-Moller J, Raastad T, Seynnes OR, Moltubakk MM, Jakobsen V, et al Mechanical properties of the patellar tendon in elite volleyball players with and without patellar tendinopathy. British journal of sports medicine. 2013;47(13): 862–868. 10.1136/bjsports-2013-092275 23833044

[33] Hopkins WG, Marshall SW, Batterham AM, Hanin J. Progressive statistics for studies in sports medicine and exercise science. Med Sci Sports Exerc. 2009;41(1): 3–13. 10.1249/MSS.0b013e31818cb278 19092709

[34] Ronnestad BR, Hansen EA, Raastad T. Effect of heavy strength training on thigh muscle cross-sectional area, performance determinants, and performance in well-trained cyclists. Eur J Appl Physiol. 2010;108(5): 965–975. 10.1007/s00421-009-1307-z 19960350

[35] Aagaard P, Andersen JL, Bennekou M, Larsson B, Olesen JL, Crameri R, et al Effects of resistance training on endurance capacity and muscle fiber composition in young top-level cyclists. Scand J Med Sci Sports. 2011;21(6): e298–307. 10.1111/j.1600-0838.2010.01283.x 21362056

[36] Taipale RS, Mikkola J, Nummela A, Vesterinen V, Capostagno B, Walker S, et al Strength training in endurance runners. Int J Sports Med. 2010;31(7): 468–476. 10.1055/s-0029-1243639 20432192

[37] Sjogaard G. Muscle morphology and metabolic potential in elite road cyclists during a season. Int J Sports Med. 1984;5(5): 250–254. 6500791 10.1055/s-2008-1025915

[38] Costill DL, Fink WJ, Pollock ML. Muscle fiber composition and enzyme activities of elite distance runners. Medicine and science in sports. 1976;8(2): 96–100. 957938

[39] Tricoli V, Lamas L, Carnevale R, Ugrinowitsch C. Short-term effects on lower-body functional power development: weightlifting vs. vertical jump training programs. J Strength Cond Res. 2005;19(2): 433–437. 15903387 10.1519/R-14083.1

[40] Wilson GJ, Newton RU, Murphy AJ, Humphries BJ. The optimal training load for the development of dynamic athletic performance. Med Sci Sports Exerc. 1993;25(11): 1279–1286. 8289617

[41] Ronnestad BR, Kvamme NH, Sunde A, Raastad T. Short-term effects of strength and plyometric training on sprint and jump performance in professional soccer players. J Strength Cond Res. 2008;22(3): 773–780. 10.1519/JSC.0b013e31816a5e86 18438241

[42] Kongsgaard M, Reitelseder S, Pedersen TG, Holm L, Aagaard P, Kjaer M, et al Region specific patellar tendon hypertrophy in humans following resistance training. Acta physiologica. 2007;191(2): 111–121. 17524067 10.1111/j.1748-1716.2007.01714.x

[43] Kubo K, Kanehisa H, Ito M, Fukunaga T. Effects of isometric training on the elasticity of human tendon structures in vivo. J Appl Physiol. 2001;91(1): 26–32. 11408409 10.1152/jappl.2001.91.1.26

[44] Kubo K, Komuro T, Ishiguro N, Tsunoda N, Sato Y, Ishii N, et al Effects of low-load resistance training with vascular occlusion on the mechanical properties of muscle and tendon. Journal of applied biomechanics. 2006;22(2): 112–119. 16871002 10.1123/jab.22.2.112

[45] Kubo K, Yata H, Kanehisa H, Fukunaga T. Effects of isometric squat training on the tendon stiffness and jump performance. Eur J Appl Physiol. 2006;96(3): 305–314. 16328192 10.1007/s00421-005-0087-3

[46] Saunders PU, Telford RD, Pyne DB, Peltola EM, Cunningham RB, Gore CJ, et al Short-term plyometric training improves running economy in highly trained middle and long distance runners. J Strength Cond Res. 2006;20(4): 947–954. 17149987 10.1519/R-18235.1

[47] Mikkola J, Rusko H, Nummela A, Pollari T, Hakkinen K. Concurrent endurance and explosive type strength training improves neuromuscular and anaerobic characteristics in young distance runners. Int J Sports Med. 2007;28(7): 602–611. 17373596 10.1055/s-2007-964849

[48] Hoff J, Helgerud J. Maximal strength training enhances running economy and aerobic endurance performance In: Hoff J, Helgerud J, editors. Football (Soccer) New Developments in Physical Training Research. Trondheim: Norwegian University of Science and Technology, Dept. of Physiology and Biomedical Engineering; 2003 p. 37–53.

[49] Noakes TD, Myburgh KH, Schall R. Peak treadmill running velocity during the VO2 max test predicts running performance. Journal of sports sciences. 1990;8(1): 35–45. 2359150 10.1080/02640419008732129

[50] Hill DW, Rowell AL. Running velocity at VO2max. Med Sci Sports Exerc. 1996;28(1): 114–119. 8775363 10.1097/00005768-199601000-00022

